# Effectiveness of Clinical Pharmacist Service on Drug-Related Problems and Patient Outcomes for Hospitalized Patients with Chronic Kidney Disease: A Randomized Controlled Trial

**DOI:** 10.3390/jcm10081788

**Published:** 2021-04-20

**Authors:** Yun-Kyoung Song, Sohyun Jeong, Nayoung Han, Heejin Na, Ha Young Jang, Minji Sohn, Yon Su Kim, Kwon-Wook Joo, Kook-Hwan Oh, Dong Ki Kim, Hajeong Lee, Jung Mi Oh, DrugTEAM Study Group

**Affiliations:** 1 College of Pharmacy and Research Institute of Pharmaceutical Sciences, Seoul National University, Seoul 08826, Korea; yksong@cu.ac.kr (Y.-K.S.); twink9114@daum.net (S.J.); hans_cp@daum.net (N.H.); hizini0701@gmail.com (H.N.); jk140117j@gmail.com (H.Y.J.); rainbowmjs@naver.com (M.S.); 2 College of Pharmacy, Daegu Catholic University, Gyeongbuk 38430, Korea; 3 Marcus Institute for Aging Research at Hebrew SeniorLife, Boston, MA 02131, USA; 4 Department of Medicine, Beth Israel Deaconess Medical Center and Harvard Medical School, Boston, MA 02131, USA; 5 College of Pharmacy, Jeju National University, Jeju-si 63243, Korea; 6 Department of Internal Medicine, Seoul National University Bundang Hospital, Seoul National University College of Medicine, Seongnam 13620, Korea; 7 Kidney Research Institute, Seoul National University College of Medicine, Seoul 08826, Korea; yonsukim@snu.ac.kr (Y.S.K.); junephro@snu.ac.kr (K.-W.J.); ohchris@hanmail.net (K.-H.O.); dkkim73@gmail.com (D.K.K.); dewyhj@hanmail.net (H.L.)

**Keywords:** pharmaceutical care service, chronic kidney disease, hospitalized patients, drug-related problems

## Abstract

(1) Background: The study aimed to analyze the effectiveness of clinical pharmacist services on drug-related problems (DRPs) and patient outcomes in inpatients with chronic kidney disease (CKD). (2) Methods: In a randomized controlled trial, the participants in the intervention group received pharmacist services, including medication reconciliation, medication evaluation and management, and discharge pharmaceutical care transition services. Participants in the control group received usual care. The primary outcome was the number of DRPs per patient at discharge. (3) Results: The baseline characteristics of 100 participants included the following: mean age, 52.5 years; median eGFR, 9.2 mL/min/1.73 m^2^. The number of DRPs in the intervention group during hospitalization increased significantly with decreasing eGFR (PR, 0.970; 95% CI, 0.951–0.989) and an increasing number of unintentional medication discrepancies at admission (PR, 1.294; 95% CI, 1.034–1.620). At discharge, the number of DRPs per patient was 0.94 ± 1.03 and 1.96 ± 1.25 in the intervention and control groups, respectively (*p* < 0.001). The service had a significant effect on the reduction of the unintentional discrepancies at discharge (*p* < 0.001). (4) Conclusion: Hospital pharmacists play an important role in the prevention of DRPs at discharge and unintentional medication discrepancies in inpatients with CKD.

## 1. Introduction

Chronic kidney disease (CKD) is defined as 3 or more months of kidney damage or an estimated glomerular filtration rate (eGFR) < 60 mL/min/1.73 m^2^ [1]. The global prevalence of CKD was estimated to be 9.1% in 2017, and increased significantly, by 87%, from 1990 to 2016, especially in elderly patients and patients with chronic diseases [2,3]. In South Korea, the number of patients diagnosed with CKD is increasing by 8.7% annually, and the average incidence of treated end-stage renal disease (ESRD) rose by 19.4 per million people annually from 2009 to 2018 [4,5]. Many risk factors, such as hypertension, diabetes, old age, and use of nephrotoxic drugs, cause CKD progression, which has contributed to a remarkably high hospitalization and renal replacement therapy (RRT) rates [1,6].

Suboptimal treatment has been reported in patients with CKD, and is associated with an increased risk of progression to ESRD. Most patients with CKD have comorbid conditions, such as electrolyte abnormalities, and cardiovascular and mineral bone disorders, and complex medication regimens [6,7]. These might increase the risk of drug-related problems (DRPs), including adverse drug reactions (ADRs), drug–drug interactions, and inappropriate dosing and blood chemistry monitoring, which result in significant morbidity and mortality and excessive cost to the healthcare system [8,9]. In particular, DRPs are common in hospitalized patients with CKD who have complex medical conditions associated with multiple comorbidities and numerous medications [10]. The concomitant multiple drugs have also been shown to impair medication adherence in patients with CKD, which might lead to disease progression and rehospitalization [6,11,12].

Due to the extraordinary comorbidity burden of CKD and associated medication complexity, the adequate management of patients with CKD requires the collaborative effort of a multidisciplinary team of nephrologists, endocrinologists, primary physicians, nurses, dieticians, and pharmacists [13,14]. Strategies to improve medication therapy management by clinical pharmacist services have the potential to contribute significantly to the multidisciplinary team to provide safe, effective, and economical care in ambulatory patients with CKD [10,14,15]. Recently, Raiisi et al. [10] conducted a systematic review that evaluated patients with CKD who received pharmacist interventions. However, among 47 studies, limited evidence from randomized controlled trials is currently available to support the benefit of clinical pharmacist services in inpatients with CKD, despite the high hospitalization rate and increasing demand for a standardized pharmacist service [3,10]. In South Korea, pharmaceutical care models for pharmacy specialists are rarely implemented as well, and tend to be limited to dispensing and drug counselling, however, the need for a standardized model among healthcare professionals has increased [16]. Therefore, the impact of clinical pharmacy services on the care of inpatients with CKD needs to be established. The aim of the present study was to analyze the effect of clinical pharmacist services on DRPs and patient outcomes in hospitalized patients with CKD in a randomized controlled trial. Patient outcomes were assessed as unintentional medication discrepancies, adherence, and acute care utilization.

## 2. Materials and Methods

### 2.1. Study Design

This was a prospective, randomized, parallel, controlled clinical trial conducted in collaboration with the nephrology team in the nephrology ward (33 beds) at Seoul National University Hospital (SNUH) in South Korea. The study participants were randomized in a 1:1 ratio to the clinical pharmacist intervention or control group using a centralized secure computer-generated program [17]. The Institutional Review Board of SNUH approved this study (IRB No. H-1511-055-719, 19 November 2015). All participants provided informed consent. All procedures were conducted in accordance with the ethical standards of the institutional and/or national research committee and with the principles of the Declaration of Helsinki.

### 2.2. Participants

Clinical pharmacists systematically identified potential participants by screening patients’ recent medical files daily. The clinical pharmacists had at least 2 years of experience with patients with CKD and were available on a full-time basis. Patients were eligible if they were (1) new admissions in the nephrology ward, (2) over 18 years of age, (3) diagnosed with CKD by their nephrologist previously, (4) confirmed to have either an eGFR of less than 60 mL/min/1.73 m^2^ or kidney damage, as shown by laboratory results in SNUH, within 3 months prior to the hospitalization, and (5) found to have one or more complications of CKD, such as hypertension, diabetes, dyslipidemia, acid-base and electrolyte disorders, anemia, mineral bone disorder, and hyperuricemia, or use one or more drugs for the treatment of these complications. Patients were excluded if they (1) were admitted for examination or procedure purposes for a short period of time, (2) did not take any medications before hospitalization, (3) had difficulties in responding to written questionnaires or interviews owing to cognitive problems, or (4) were participating in other studies. Those who met the above criteria were considered eligible and were invited to participate in the study. Consent for participation was obtained from all patients.

### 2.3. Intervention

**Figure 1:**
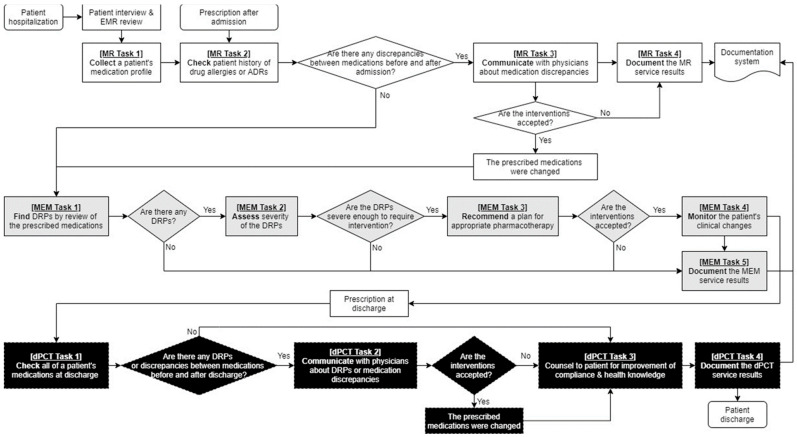
Drug Therapy Evaluation and Management (DrugTEAM) service model for hospitalized patients with chronic kidney disease. Abbreviation: ADR, adverse drug reaction; DRP, drug-related problem; EMR, electronic medical record; MEM, medication evaluation and management; MR, medication reconciliation; dPCT, discharge pharmaceutical care transition.

In the clinical pharmacist intervention group, the hospitalized participants received the collaborative multidisciplinary drug therapy evaluation and management (DrugTEAM) service based on the internal guidelines for inpatients [18,19]. Clinical pharmacists were the service provider to patients and healthcare professionals, and the intervention was augmented by the DrugTEAM service model based on the algorithm presented in Figure 1. They conducted a structured, patient-centered medication review, communicated with healthcare professionals and patients, and documented inpatients diagnosed with CKD daily. Briefly, the service included a (1) medication reconciliation (MR) service to reduce discrepancies in medicines prescribed within 24 h after admission compared with those in medicines prescribed before admission, (2) medication evaluation and management (MEM) service to promote the appropriateness of the pharmacotherapy, and (3) discharge pharmaceutical care transition (dPCT) service to reduce the medication discrepancies before and after discharge and improve patient compliance and health knowledge. The MR service included collecting, checking, communicating, and documenting a patient’s medication history. The MEM service consisted of finding, assessing, recommending, monitoring, and documenting the prescribed drugs. In the dPCT services, the prescribed drugs were collected, checked, communicated, and documented at discharge and the patient and caregiver were counselled about medicines using educational materials, such as written medication guides, timetables, pillboxes, and medication diaries.

Participants in the control group received usual care from pharmacists and physicians, without implementing DrugTEAM service model. The usual care mainly consisted of dispensing prescribed drugs and discharge education on the safe and appropriate use of the medicines. In Korea, medication review by pharmacist is neither part of the usual care nor reimbursed [20].

### 2.4. Outcome Measures

The primary outcome was the average number of DRPs per patient at discharge. The DRPs were classified into the following subitems: prescription without indication, indication without prescription, duplicated prescription, inappropriate drug selection, inappropriate dosage/administration, allergy, ADR, drug interaction, nonadherence, cost issue, and others [21,22]. Secondary outcomes were (1) medication adherence for discharge drugs, measured using the Modified Morisky Scale (MMS), which consists of questions that assess motivation and knowledge at the first outpatient visit after discharge [23], (2) a composite of acute care utilization (unexpected hospitalization or emergency center visit) within 3 months of discharge, and (3) change in the number of unintentional medication discrepancies at discharge compared with that at the time of admission. Unintentional discrepancies were defined as erroneous and unjustified medication discordance between medication use history and prescribed medicines at the time of hospitalization or discharge, which included medicine omission, addition, substitution, and duplication, or any changes in regimen, administration route, and drug formulation [24]. We also assessed (4) the DRPs during hospitalization and their resolution rate by the pharmacist’s intervention to evaluate the outcomes of the MEM service.

Both groups had the same assessments (including laboratory testing) at admission, discharge, the first visit after discharge, and 3 months after discharge. Prospective assessment was performed in the intervention group during hospitalization. However, the DRPs and medication discrepancies were retrospectively evaluated in the control group.

### 2.5. Statistical Analysis

In a randomized controlled trial by Lenander et al. [25], ambulatory patients had a mean of 1.73 ± 0.63 DRPs per patient at baseline. With a sample size of 50 patients in each group, and assuming α of 0.05, we estimated that we had 90% power to detect an 23% reduction in the number of DRPs between the intervention and control group with 10% dropout rate [8,25].

Data are shown as numbers and percentages for categorical variables, means and standard deviation (SD) for continuous parametric data, and medians and interquartile range (IQR) for nonparametric variables. Fisher’s exact and chi-square tests were used to compare categorical data and unpaired t and Mann–Whitney tests were used to compare continuous data. The percent change in unintentional medication discrepancies from admission to discharge in each group was compared using the Wilcoxon signed-rank test. Poisson regression analysis was used to study the risk factors for the occurrence of DRPs during hospitalization. Statistical significance was set at a two-sided *p-*value < 0.05, and data analysis and computation were conducted using SAS version 9.4 (SAS Institute, Cary, NC, USA). Patients excluded from the study were followed up until their withdrawal unless they specifically allowed longer follow-up.

## 3. Results

### 3.1. Demographic Characteristics

**Figure 2:**
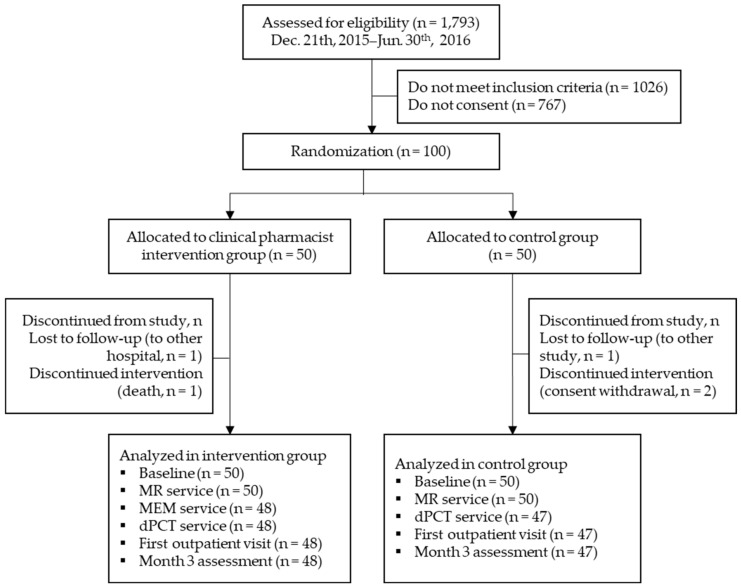
CONSORT flow diagram of randomization and patient participation. Abbreviation: MEM, medication evaluation and management; MR, medication reconciliation; dPCT, discharge pharmaceutical care transition.

Patients were recruited from 19 December 2015 to 31 May 2016, monitored until discharge (median period of hospitalization was 4 days; IQR, 3–8 days), and followed up at the first outpatient visit after discharge (median period until the first visit after discharge was 9 days; IQR, 6.5–14 days). Of the 1793 patients screened, only 100 fulfilled the inclusion criteria and participated in the study (50 were randomly assigned to the clinical pharmacist intervention group and 50 were assigned to the control group). Five patients were excluded owing to follow-up loss, consent withdrawal, or death, and 95 participants completed the follow-up visit after discharge (Figure 2).

The baseline characteristics of the study participants were similar in both groups (Table 1). The mean age of the patients was 52.5 years, and 60% were men. The median value of the eGFR was 9.2 mL/min/1.73 m^2^ (IQR, 5.6–20.8 mL/min/1.73 m^2^). At baseline, 65% of patients were diagnosed with stage 5 CKD, and 50% were undergoing RRT. Patients had a median of five comorbidities (IQR, 4–6) and received nine different drugs (IQR, 7–12) within 24 h after the admission with an average adherence score of 4.5, as measured by MMS.

### 3.2. Drug-Related Problems by the DrugTEAM Service

Participants in the intervention group were prescribed a median of 12.5 drugs (IQR, 9.75–15) during hospitalization in the nephrology ward. In total, 182 DRPs were identified by the MEM service for inpatients with CKD (Table 2 and Table S1). At least one DRP was recorded for each participant in the intervention group, and the median number of DRPs per patient was 3.5 (IQR, 2–5). The majority of the DRPs were recorded in the domains of indication without prescription (65, 35.7%) and inappropriate dosage/administration (58, 31.9%). Twenty prescriptions (11.0%) had problems with no indications, and problems with inappropriate drug prescription (12, 6.6%) or ADRs (11, 6.0%) were often recorded. Additionally, problems frequently occurred with drugs for the management of comorbidities, such as mineral bone disorder (36, 19.8%), hypertension/cardiovascular diseases (34, 18.7%), and anemia (24, 13.2%). Of the DRPs, 149 (81.9%) were completely solved by physicians through 197 pharmacist interventions, which included recommendations for new drugs for the management of comorbid diseases (73, 37.1%), discontinuation of medication according to symptoms or electrolyte/blood pressure levels (58, 29.4%), and dosage adjustment according to eGFR or therapeutic dose monitoring (TDM) results (45, 22.8%; Table 3). The number of DRPs increased significantly with decreasing eGFR (prevalence ratio (PR), 0.970; 95% confidence interval (CI), 0.951–0.989) and an increasing number of unintentional medication discrepancies at admission (PR, 1.294; 95% CI, 1.034–1.620) during a median of 5 days of hospitalization (IQR, 3–8 days; Table 4). Other factors, such as age, sex, implementation of RRT at admission, number of comorbid diseases, medication adherence at admission, and number of medicines per patient at admission and during hospitalization, were not significantly associated with the incidence of DRPs.

At discharge, the median number of prescribed drugs per patient was significantly lower in the intervention group (9) than in the control group (11; *p* = 0.005). The primary outcome, the number of DRPs per patient at discharge, was significantly affected by the clinical pharmacist intervention (Table 5). Forty-five DRPs occurred in 54.2% of participants in the intervention group and 91.5% (92) in the control group at discharge. The number of DRPs per patient was 0.94 ± 1.03 and 1.96 ± 1.25 in the intervention and control group, respectively (*p* < 0.001). We observed a nonsignificant difference in the types of DRP between the groups. The main problems at discharge in each group were indication without prescription (31.1% in the intervention group vs. 39.1% in the control group) and inappropriate dosage/administration (33.3% in the intervention group vs. 20.7% in the control group). The DRPs according to the pharmacotherapy indications at discharge were similar to those during hospitalization, but only 23 of 45 DRPs (51.1%) that occurred in the intervention group were resolved at discharge (Table S1 and Table 3).

### 3.3. Adherence and Acute Care Utilization after Discharge by the DrugTEAM Service

The medication adherence score measured by MMS at the first outpatient visit after discharge was higher in the intervention group (5.2 ± 1.0) than in the control group (4.9 ± 1.3), but not significantly so (*p* = 0.205; Table 5). After the DrugTEAM service in the intervention group, 39 patients (81.3%) had an increased MMS score after discharge compared with during hospitalization, but this improvement was not significantly different to that in the control group, in which the MMS score increased in 30 patients (63.8%; *p* = 0.057). The number of patients using acute care within 3 months of discharge was 16 (33.3%) and 12 (25.5%) in the intervention and control groups, respectively, and the difference was not statistically significant (*p* = 0.404). Among 50 patients who received RRT at baseline, seven patients in the intervention group (35.0%) and eight patients in the control group (26.7%) visited the emergency centers or hospitalized unexpectedly. The clinical pharmacist service for these patients did not affect the acute care utilization after discharge (OR, 0.675; 95% CI, 0.199–2.298, data not shown).

### 3.4. Unintentional Medication Discrepancies by the DrugTEAM Service

**Figure 3:**
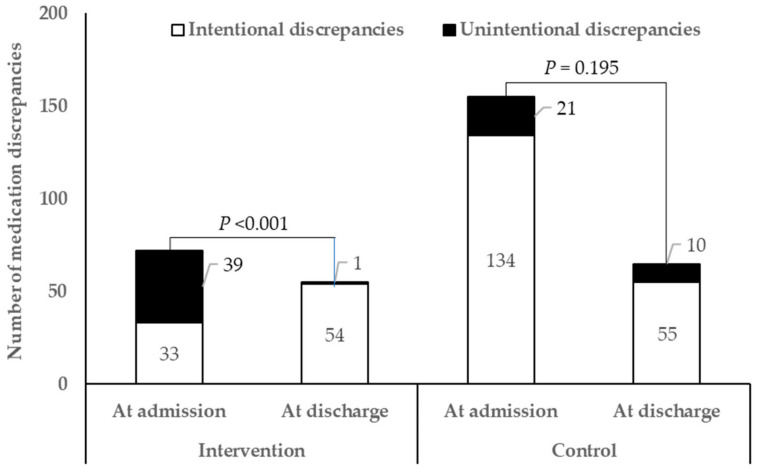
Changes in medication discrepancies at discharge compared to hospitalization in clinical pharmacist intervention and control groups.

Changes in medication discrepancies at discharge compared with during hospitalization in each group are shown in Figure 3. Unlike the control group (21 unintentional discrepancies at admission and 10 at discharge, *p* = 0.195), the clinical pharmacist service for inpatients with CKD had a significant effect on the reduction of unintentional discrepancies at discharge (39 unintentional discrepancies at admission and one at discharge, *p* < 0.001). Most unintentional medication discrepancies (52/71, 73.2%) occurred as erroneous omissions of previously administered medicines or the addition of new drugs.

## 4. Discussion

Hospitalized patients with CKD have a high risk of drug duplication, interactions, and adverse events, which could result in extended hospital stays, higher costs, and low quality of life. In this randomized controlled trial, we established that the clinical pharmacist intervention, based on comprehensive medication review, patient counselling, and interprofessional communication through MR, MEM and dPCT services, can reduce the number of DRPs, as well as unintentional medication discrepancies, in hospitalized patients with CKD longitudinally from admission to up to 3 months after discharge. The DrugTEAM service model was developed as a collaborative multidisciplinary team care model to improve the clinical outcomes of pharmacotherapy by reducing the number of DRPs and increasing medication adherence [18]. We adopted this model to standardize clinical pharmacist service processes for inpatients with CKD in Korea. To the best of our knowledge, this is the first randomized controlled study to assess the potential role of clinical pharmacist services in hospitalized patients with CKD [10].

In this study, two-thirds of the participants had stage 5 CKD with an average eGFR of 8.9 mL/min/1.73 m^2^; thus, this included more patients with advanced CKD than similar studies in outpatients or inpatients with CKD not treated by dialysis [8,26]. It has been reported that Korean patients with CKD are generally younger than patients from other ethnicities in International Network of Chronic Kidney Disease cohorts [27]. These patients were younger, on average, than those in the related studies, which might affect the differences in comorbidity conditions, number of medications, and medication adherence score [8,26,28]. The number of prescribed drugs increased to a median 12.5 during hospitalization from 8 at admission in the intervention group. Various surveys have also shown that patients on dialysis are prescribed an average of 10–12 medications per day, and some patients take 20–30 doses each day [14]. Considering the high pill burden and its contribution to frequent DRPs and poor medication adherence in this patient population, medication review by trained pharmacy practitioners might contribute to the positive outcomes of pharmacotherapy in multidisciplinary CKD teams [14,29].

The majority of clinical pharmacy practice processes, often labeled as interventions, include medication review to identify any DRPs [14]. A median of 3.5 DRPs occurred per inpatient with CKD in the study, whereas outpatients with CKD in Canada experience only an average of 2.2 DRPs [8]. The higher DRPs found in this study might be attributable to inclusion of inpatients with CKD with generally complex clinical conditions, and many of them on dialysis unlike the Canadian study, in which only patients with CKD stages 3–4 were included in outpatient setting.

The well-known main pharmacist interventions are new pharmacotherapy recommendations, dosage adjustments according to kidney function, the monitoring of laboratory parameters, and the assessment of the appropriateness of medications prescribed at each point of care; these were mainly undertaken by pharmacists for patients in the intervention group [10,14]. Low eGFR and unintentional medication discrepancies at admission affected the number of DRPs in hospitalized patients with CKD in this study. This is consistent with the results of the previous studies that CKD severity and medication errors are associated with medication-related problems [30,31]. However, well-known risk factors for DRPs, such as polypharmacy and comorbidity, were not significantly associated with DRPs in this study, possibly owing to the relatively small sample size and the propensity of bias towards patients with CKD stage 5 with high medication burdens and complicated comorbidity conditions [32,33]. The present study demonstrated that clinical pharmacist interventions significantly reduced the number of DRPs at discharge compared with usual care. The acceptance rate by medical team for the pharmacist-driven proposed changes was 81.9% during hospitalization, which indicates a high level of acceptability compared with similar studies in patients with CKD [34]. However, as most DRPs were resolved by the pharmacist intervention during hospitalization, approximately half of the interventions for DRPs at discharge were not accepted by physicians. It was reported that among 897 DRPs identified in 442 patients with CKD, 47.6% were classified as DRPs with mild severity, and the pharmacist service contributed to the reduction of new DRPs after 12 months [31]. Therefore, it is considered that the low incidence of new DRPs and resolution of potentially important DRPs during hospitalization in the intervention group might have contributed to the low acceptance rate of DRP interventions at discharge. DRPs have been associated with negative health outcomes, and it is anticipated that these pharmacist interventions and their high acceptance rates will improve clinical outcomes associated with anemia, diabetes, hyperlipidemia, hypertension, and secondary hyperparathyroidism in patients with CKD [10,32].

In this study, the DrugTEAM service, which include patient counselling at discharge nominally, improved the medication adherence score compared with that in the control group at the first outpatient visit, but not significantly. The relatively young participants and their disease severity might give them alertness in taking medications and might demonstrate insignificant results. However, since the complicated regimen and polypharmacy are one of the detrimental factors in patient care, it is expected that medication adherence can be enhanced by multimodal, ongoing patient counselling and mapping medication routines in everyday activities, not by one-time medication education, as performed in this study [11,35]. Moreover, the results might have been influenced by the lack of reliability of the MMS, which was developed by modifying the original four-item Morisky Scale [36,37]. Joost et al. [35] demonstrated enhanced medication adherence measured by days with correct drug dosing in kidney transplant recipients, but no difference between intensified and standard care groups when medication adherence was measured by MMS. Additionally, there was no statistical difference in acute care utilization between the groups, but incidence in the acute care utilization was higher in intervention group (33.3%) compared to control (25.5%). In a previous study, an intervention for medication therapy management by pharmacists during the transition from hospital to home did not reduce their high rate of acute care utilization, such as hospitalization or emergency department and urgent care center visits, for 90 days after hospital discharge [26]. Moreover, the high proportion of dialysis patients in control group (40% vs. 26%) might enable them to visit hospitals regularly reducing their acute emergency care utilization. Therefore, it is necessary to establish a system that incorporates an in-hospital multifaceted clinical pharmacist intervention based on medication review, a structured patient interview using a motivational interview approach for at least 30 min, and post-discharge follow-up for hospitalized patients [38].

Medication errors can be attributed to discrepancies in medication history at the time of care transition. Therefore, the MR service is an important component of a mediation safety program for patients with advanced-stage CKD [39]. It has been reported that approximately 34% of medication discrepancies are potentially harmful [24]. The DrugTEAM service during hospitalization contributed to a significant reduction in such unintentional discrepancies at discharge compared with the usual care group, which is a consistent with previous results in kidney transplantation patients [12]. Therefore, medication reconciliation should be performed at every patient visit, including routine care and transitions of care before the comprehensive medication review, by pharmacists to reduce the risk of DRPs and the associated consequences.

As experts in medication management with a close relationship with patients with chronic diseases, pharmacists are well-positioned to identify patients with CKD, reduce the cardiovascular risk, assist in disease management, and produce substantial cost savings [15,40]. Due to the complexity of the disease and evidence of the positive role of pharmacists in patients with CKD worldwide, the Korean government has reimbursed multidisciplinary professionals, including physicians, nurses, pharmacists, and nutritionists, to educate patients with CKD since 2018 [20]. It is necessary to develop this multidisciplinary team care model to actively involve pharmacists in TDM and DRP management for better pharmacotherapy outcomes in inpatients with CKD.

This study has some limitations that need to be considered. Firstly, this study was performed in a single tertiary hospital, although a statistically significant number of participants were recruited from the center. Multicenter studies with large sample sizes are needed to analyze the various clinical outcomes of pharmacist services in hospitalized patients. Secondly, the study could not be entirely blinded because the intervening pharmacist and patient knew the result of the allocation due to the educational nature of this study. We used a blinded randomization procedure and blinded the pharmacists who performed the assessment at admission, discharge, and post-discharge to avoid different levels of diligence in the review. Finally, because of the requirements for informed consent, patients with severe dementia and delirium were underrepresented in our study population. Cognitive disorders and dementia are prevalent among patients with stage 5 CKD, which accounted for approximately 60% of the participants in this study. Therefore, the effectiveness of our intervention in cognitively impaired inpatients with CKD is unknown.

## 5. Conclusions

In conclusion, hospital pharmacists could play an important role in the prevention of DRPs at discharge and unintentional medication discrepancies in patients with CKD. This represents a promising approach for medication management in hospitalized patients with CKD by clinical pharmacists based on the DrugTEAM service model, which could have major public health implications.

## Figures and Tables

**Table 1:** Baseline characteristics.

Characteristics	Total (*n* = 100)	Intervention Group (*n* = 50)	Control Group (*n* = 50)	*p* Value
Age, mean ± SD, years	52.5 ± 16.9	51.0 ± 16.6	54.0 ± 17.4	0.381
Age, median (IQR), years	55 (37–67)	51 (36.3–64)	57.5 (38–67)	
Male, *n* (%)	60 (60)	31 (62)	29 (58)	0.683
eGFR, median (IQR), mL/min/1.73 m^2^	9.2 (5.7–20.8)	8.9 (5.9–20.6)	9.2 (5.4–20.9)	0.834
CKD stage, *n* (%)				
Stage 2 (eGFR 60–89 mL/min/1.73 m^2^)	3 (3)	2 (2)	1 (2)	1.000
Stage 3 (eGFR 30–59 mL/min/1.73 m^2^)	12 (12)	7 (14)	5 (10)	0.538
Stage 4 (eGFR 15–29 mL/min/1.73 m^2^)	20 (20)	7 (14)	13 (26)	0.134
Stage 5 (eGFR < 15 mL/min/1.73 m^2^)	65 (65)	34 (68)	31 (62)	0.529
Renal replacement therapy, *n* (%)				
Dialysis	33 (33)	13 (26)	20 (40)	0.137
Kidney transplantation	17 (17)	7 (14)	10 (20)	0.425
None	50 (50)	30 (60)	20 (40)	0.046
CKD etiology, *n* (%)				
Diabetes mellitus	28 (28)	12 (24)	16 (32)	0.373
Hypertension	8 (8)	5 (10)	3 (6)	0.715
Glomerulonephritis	40 (40)	23 (46)	17 (34)	0.221
Others	9 (9)	5 (10)	4 (8)	1.000
Unknown	15 (15)	5 (10)	10 (20)	0.161
Diagnosis at admission, *n* (%)				
Uremia	33 (33)	17 (34)	16 (32)	0.832
Renal replacement therapy	26 (26)	11 (22)	15 (30)	0.362
Infection	16 (16)	9 (18)	7 (14)	0.585
Graft rejection	8 (8)	3 (6)	5 (10)	0.715
Cardiovascular diseases	7 (7)	5 (10)	2 (4)	0.436
Others	10 (10)	5 (10)	5 (10)	1.000
Number of comorbid diseases, median (IQR)	5 (4–6)	5 (2–9)	5 (5–7)	0.009
Adherence at admission measured by MMS, mean ± SD	4.5 ± 1.3	4.1 ± 1.5	4.5 ± 1.5	0.226
Number of medicines per patient at admission, median (IQR)	9 (7–12)	8 (5.8–12)	9 (8–12)	0.241

Abbreviation: CKD, chronic kidney disease; eGFR, estimated glomerular filtration rate; IQR, interquartile range; MMS, Modified Morisky Scale.

**Table 2:** Drug-related problems (DRPs) in an intervention group implemented medication evaluation and management (MEM) service during hospitalization (*n* = 48).

Outcomes	DRPs, *n* (%)	Resolved DRPs, *n* (%)
Patients with any DRPs during hospitalization, *n* (%)	48 (100.0)	NA
Total number of DRPs during hospitalization	182 (100.0)	149 (81.9)
Number of DRPs per patient during hospitalization, mean ± SD	3.8 ± 1.8	NA
DRP classification ^a^, *n* (%)		
Prescription without indication	20 (11.0)	20 (100.0)
Indication without prescription	65 (35.7)	53 (81.5)
Duplicated prescription	2 (1.1)	2 (100.0)
Inappropriate drug selection	12 (6.6)	9 (75.0)
Inappropriate dosage/administration	58 (31.9)	49 (84.5)
Allergy	2 (1.1)	2 (100.0)
Adverse drug reaction	11 (6.0)	7 (63.6)
Cost issue	4 (2.2)	3 (75.0)
Others	8 (4.4)	4 (50.0)

Abbreviation: NA, not applicable. ^a^ No DRP was found in the categories of drug interaction and nonadherence.

**Table 3:** Planned interventions for each drug-related problem (DRP) in intervention group implemented clinical pharmacist service during hospitalization.

Planned Interventions	MEM Service, *n* (%)	dPCT Service, *n* (%)
Drug started	73 (37.1)	20 (37.0)
Drug paused or stopped	58 (29.4)	16 (29.6)
Dosage increased	19 (9.6)	0
Dosage decreased	26 (13.2)	8 (14.8)
Instructions for use changed	9 (4.6)	1 (1.9)
Administration time changed	2 (1.0)	3 (5.6)
Information of insurance coverage provided	4 (2.0)	2 (3.7)
Laboratory test recommended for drug efficacy monitoring	6 (3.0)	3 (5.6)
Others	0	1 (1.9)
Total	197 (100.0)	54 (100.0)

Abbreviation: dPCT, discharge pharmaceutical care transition; MEM, medication evaluation and management.

**Table 4:** Risk factors for drug-related problems (DRPs) in an intervention group implemented medication evaluation and management (MEM) service during hospitalization.

Factors	Prevalence Ratio (95% CI)	*p* Value
Age	0.989 (0.973–1.006)	0.223
Gender (male)	1.248 (0.742–2.009)	0.404
Estimated glomerular filtration rate	0.970 (0.951–0.989)	0.002
Renal replacement therapy		
None	Reference	
Dialysis	0.839 (0.469–1.502)	0.555
Kidney transplantation	0.979 (0.444–2.160)	0.958
Number of comorbid diseases	0.924 (0.778–1.097)	0.368
Adherence measured by MMS at admission	0.979 (0.809–1.183)	0.823
Number of medicines per patients at admission	0.936 (0.864–1.014)	0.105
Number of medicines per patients during hospitalization	0.976 (0.904–1.054)	0.534
Number of unintentional medication discrepancies at admission	1.294 (1.034–1.620)	0.025

Abbreviation: CI, confidence interval; MMS, Modified Morisky Scale.

**Table 5:** Drug-related problems (DRPs) and patient outcomes of a discharge pharmaceutical care transition (dPCT) service.

Outcomes		Intervention (*n* = 48)	Control (*n* = 47)	*p* Value
DRPs	Patients with any DRPs at discharge, *n* (%)	29 (60.4)	43 (91.5)	<0.001
	Total number of DRPs at discharge	45	92	<0.001
	Number of DRPs per patient at discharge, mean ± SD	0.9 ± 1.0	2.0 ± 1.3	<0.001
	DRP classification ^a^, *n* (%)			
	Prescription without indication	1 (2.2)	8 (8.7)	0.271
	Indication without prescription	14 (31.1)	36 (39.1)	0.360
	Inappropriate drug selection	5 (11.1)	4 (4.3)	0.154
	Inappropriate dosage/administration	15 (33.3)	19 (20.7)	0.107
	Adverse drug reaction	3 (6.7)	10 (10.9)	0.545
	Drug interaction	0	8 (8.7)	0.053
	Cost issue	2 (4.4)	4 (4.3)	1.000
	Others	5 (11.1)	3 (3.3)	0.114
Adherence	MMS at first visit after discharge, mean ± SD	5.2 ± 1.0	4.9 ± 1.3	0.205
	Patients with increased MMS score, *n* (%)	39 (81.3)	30 (63.8)	0.057
Acute care utilization	Patients readmitted or visiting emergency center within 3 months of discharge, *n* (%)	16 (33.3)	12 (25.5)	0.404

Abbreviation: MMS, Modified Morisky Scale; SD, standard deviation. ^a^ No DRP was found in the categories of duplicated prescription, allergy and nonadherence.

## Supplementary Materials

The following are available online at https://www.mdpi.com/article/10.3390/jcm10081788/s1, Table S1: Drug-related problems (DRPs) according to pharmacotherapy indications in intervention group implemented clinical pharmacist service during hospitalization.
