# Supplementary material for: Effectiveness of Clinical Pharmacist Service on Drug-Related Problems and Patient Outcomes for Hospitalized Patients with Chronic Kidney Disease: A Randomized Controlled Trial
Source: J Clin Med. 2021 Apr 20;10(8):1788. doi: 10.3390/jcm10081788 (PMC8072770; doi:10.3390/jcm10081788)
Supplement: Supplementary file 1 [file jcm-10-01788-s001.zip › jcm-1172703-supplementary.pdf]

Table S1. Drug-related problems (DRPs) according to pharmacotherapy indications in intervention group implemented clinical pharmacist service during hospitalization.

Indication	MEM service		dPCT service		Main contents of indication-specific recommendation by clinical pharmacists
	DRPs, n (%)	Resolved DRPs, n (%)	DRPs, n (%)	Resolved DRPs, n (%)	
Mineral bone disease	36 (19.8)	31 (86.1)	7 (15.6)	3 (42.9)	- Addition, discontinuation, or dosage adjustment of phosphate–binding agents, vitamin D or calcimimetics according to blood phosphorus, calcium, PTH, or 25(OH)D levels;<br>- Change the administration time of phosphate–binding agents;<br>- Blood test for the evaluation of phosphate–binding agents or calcimimetics use;<br>- Dosage reduction of phosphate–binding agents based on the maximum daily dose of calcium.
Hypertension/ cardiovascular disorders	34 (18.7)	28 (82.4)	5 (11.1)	2 (40.0)	- Addition, discontinuation, or dosage adjustment of antihypertensive drug therapy according to the blood pressure level;<br>- Change the administration time of antithrombotic agents owing to surgery or procedure.
Anemia	24 (13.2)	18 (75.0)	7 (15.6)	3 (42.9)	- Blood test for the evaluation of ESA, or iron preparation use;<br>- Start, or discontinue ESA or iron supplement based on the hemoglobin levels, transferrin saturation or serum ferritin levels;<br>- Dosage adjustment of ESA according to hemoglobin level.
Hyperuricemia	13 (7.1)	11 (84.6)	4 (8.9)	3 (75.0)	- Addition, discontinuation, or dosage adjustment of xanthine oxidase inhibitors based on the serum uric acid or CrCl levels;<br>- Discontinue colchicine based on patient symptoms.
Gastrointestinal disorders	15 (8.2)	12 (80.0)	1 (2.2)	0	- Start laxatives for the prevention or treatment of constipation;<br>- Start, or discontinue acid suppression therapy, promotility agents or digestive enzymes;<br>- Start proton-pump inhibitors owing to high-dose corticosteroid use;<br>- Renal dosing of histamine H_2 antagonists.
Metabolic acidosis	11 (6.0)	11 (100.0)	0	NA	- Addition, discontinuation, or dosage adjustment of sodium bicarbonate based on plasma total CO_2 level.
Infections	8 (4.4)	8 (100.0)	3 (6.7)	3 (100.0)	- Renal dosing of antimicrobial agents.
Fluid retention	8 (4.4)	6 (75.0)	2 (4.4)	0	- Start or discontinue diuretics based on patient symptoms such as oedema or urine output;<br>- Change thiazide diuretics to loop diuretics in patients with CrCl <30 mL/min;<br>- Hold diuretics before a coronary angioplasty procedure.
Dyslipidemia	6 (3.3)	3 (50.0)	8 (17.8)	5 (62.5)	- Addition or dosage adjustment of an HMG–CoA reductase inhibitor based on blood lipoprotein concentrations.
Nutritional disorders	6 (3.3)	4 (66.7)	1 (2.2)	0	- Start multivitamins with an insurance coverage in patients receiving dialysis.
Disorders of electrolyte	5 (2.7)	5 (100.0)	0	NA	- Addition, discontinuation, or dosage adjustment of potassium chloride or a potassium-lowering agent

homeostasis					according to the blood potassium level.
Hyperglycemia	3 (1.6)	3 (100.0)	0	NA	- Change to antidiabetic drugs that do not require dosage adjustment according to kidney function;<br>- Resume or discontinue metformin according to S_{cr} level.
Immune disorders	3 (1.6)	2 (66.7)	1 (2.2)	NA	- Blood test for TDM of calcineurin inhibitors;<br>- Discontinuation or dosage adjustment of calcineurin inhibitors according to TDM.
Neurological disorders	2 (1.1)	1 (50.0)	1 (2.2)	0	- Renal dosing of pregabalin or gabapentin.
Others	8 (4.4)	6 (75.0)	5 (11.1)	4 (80.0)	- Discontinue adsorptive carbon based on S_{cr} level owing to insurance coverage;<br>- Start or discontinue dermal corticosteroids or oral antihistamines based on the symptom of pruritus;<br>- Renal dosing of histamine H_1 antagonists or analgesics.
Total	182 (100.0)	149 (81.9)	45 (100.0)	23 (51.1)	

Abbreviation: CrCl, creatinine clearance; dPCT, discharge pharmaceutical care transition; ESA, erythropoiesis-stimulating agents; MEM, medication evaluation and management; NA, not applicable; PTH, parathyroid hormone; Scr, serum creatinine; TDM, therapeutic dose monitoring.
